# Injection of Fluoro-Gold into the tibial nerve leads to prolonged but reversible functional deficits in rats

**DOI:** 10.1038/s41598-019-46285-7

**Published:** 2019-07-09

**Authors:** Daguo Mi, Ying Yuan, Yanping Zhang, Jiahui Niu, Yaxian Wang, Junying Yan, Yumin Yang, Wen Hu

**Affiliations:** 1 Department of Orthopedics, Nantong Hospital of Traditional Chinese Medicine, Nantong, Jiangsu 226001 China; 2 Key Laboratory for Neuroregeneration of Ministry of Education and Co-innovation Center for Neuroregeneration of Jiangsu Province, Nantong University, Nantong, Jiangsu 226001 China; 3 The Affiliated Hospital of Nantong University, Nantong, Jiangsu 226001 China; 4 School of Medicine, Nantong University, Nantong, Jiangsu 226001 China; 5 Present Address: Department of Burns and Plastic Surgery and Cosmetology, Longyan First Hospital, Longyan, Fujian, 364000 China; 6 Present Address: Department of Neurochemistry, Inge Grundke-Iqbal Research Floor, New York State Institute for Basic Research in Developmental Disabilities, Staten Island, NY 10314 USA

**Keywords:** Somatic system, Rat

## Abstract

Tract tracing with neuronal tracers not only represents a straightforward approach to identify axonal projection connection between regions of the nervous system at distance but also provides compelling evidence for axonal regeneration. An ideal neuronal tracer meets certain criteria including high labeling efficacy, minimal neurotoxicity, rapid labeling, suitable stability *in vivo*, and compatibility to tissue processing for histological/immunohistochemical staining. Although labeling efficacy of commonly used fluorescent tracers has been studied extensively, neurotoxicity and their effect on neural functions remains poorly understood. In the present study, we comprehensively evaluated motor and sensory nerve function 2–24 weeks after injection of retrograde tracer Fluoro-Gold (FG), True Blue (TB) or Fluoro-Ruby (FR) in the tibial nerve in adult Spague-Dawley rats. We found that motor and sensory nerve functions were completely recovered by 24 weeks after tracer exposure, and that FG lead to a more prolonged delay in functional recovery than TB. These findings shed light on the long-term effect of tracers on nerve function and peripheral axonal regeneration, and therefore have implications in selection of appropriate tracers in relevant studies.

## Introduction

Tract tracing with neuronal tracers provides compelling evidence for identification of anatomical axonal projection and characterization of integrity/restoration of axonal connection^1,2^. Neuronal tracers employed in neuroanatomy and neuroregeneration studies mainly include fluorescent molecules, fluorophore or biotin-conjugated dextran, horseradish peroxidase and derivatives, carbocyanine dyes, cholera toxin subunit B-saporin, and viral vectors carrying a reporter DNA sequence^3–8^. Among others, fluorescent retrograde tracers are popular in neuroscience studies since tracer exposure in the nerve or axonal pathway distal to injury provides a unique opportunity to directly visualize neurons which have regenerated axons across the injury site^2,9–11^. Labeling efficacy of retrograde fluorescent tracers has been extensively studied^12–16^; however, toxicity of tracers to the labeled neurons/axons, which is another important property to be considered, was seldom investigated and poorly understood^17–20^.

Neurotoxicity of tracers can be exhibited as pathological alterations, deficits in physiological function of neurons/axons, or both^18,21^. We recently found that injection of retrograde tracers Fluoro-Gold (FG), True Blue (TB) and Fluoro-Ruby (FR) in the tibial nerve in rats resulted in varying levels of axonal degeneration and functional impairment: FG lead to chemical severance of the nerve, and thus resulted in functional deficits almost as acute and complete as that caused by surgical transection of the nerve; TB also caused functional impairment and axonal degeneration distal to injection, but to a lesser degree than FG; whereas the effect of intra-neural injection of FR on nerve function and axonal integrity was negligible^21^. In the present study, we further investigated how tracer-induced functional deficits evolve over time by using a battery of well-established functional assessments, and we found that motor and sensory nerve functions were completely recovered by 24 weeks after injection of tracers in the tibial nerve, despite a prolonged delay in the case of FG. We utilized FR as a reference, as this tracer did not cause significant function deficits after injected even at a greater amount into the tibial nerve, similar to the vehicle control (saline)^21^.

## Results

### Both FG and TB cause reversible impairment in motor/sensory nerve function

**Figure 1:**
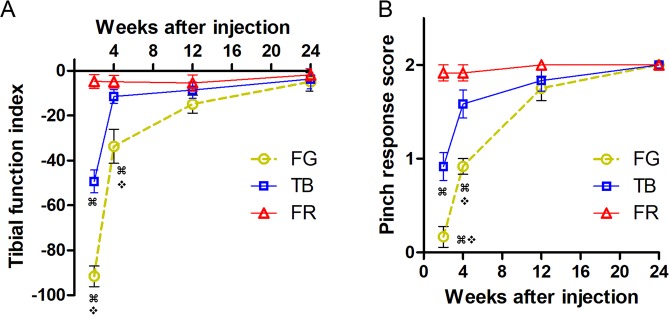
Motor and sensory deficits resulted from fluorescent tracers are reversible but FG impedes functional recovery for a more prolonged period than TB. (**A**) Walking track analysis showing tibial function index over time. (**B**) Score of pinch response in the fifth toe. Data are expressed as mean±SEM (n = 12 rats/group unless otherwise specified) and analyzed with repeated measures analysis of variance (ANOVA) followed by Bonferroni’s *post hoc* comparisons for data of all time points excluding 24 weeks after injection where data of only 6 animals per group were available. ^⌘^*P* < 0.001 compared to FR, ^❖^*P* < 0.001 compared to TB.

Walking track analysis, in which a sciatic/tibial/peroneal function index is calculated, is a well established assessment of motor function of the rodent sciatic/tibial/peroneal nerve after injury and repair^22,23^. We performed walking track analysis at 2, 4, 12 and 24 weeks after injection of tracers. We found that similar to that observed previously^21^, FR did not induce a significant motor deficit after injection into the tibial nerve; the TB group recovered TFI to a nearly normal level by 4 weeks after injection (Fig. 1A). However, the FG group showed more pronounced impairment, despite a clear trend of recovery, in motor function by 4 weeks after injection, and complete recovery by 24 weeks after tracer application (Fig. 1A).

We employed toe pinch reflex, a simple but reliable assessment of digital cutaneous sensibility^24,25^, to evaluate sensory function in the volar aspect of the fifth toe, an autonomous region of the tibial nerve. Scoring of the pinch response showed a similar recovery pattern as seen in tibial function index (Fig. 1B), indicating that FG leads to more prolonged yet still reversible sensory deficit when injected in the peripheral nerve.

### FG causes prolonged electrophysiological deficit in the target muscle

**Figure 2:**
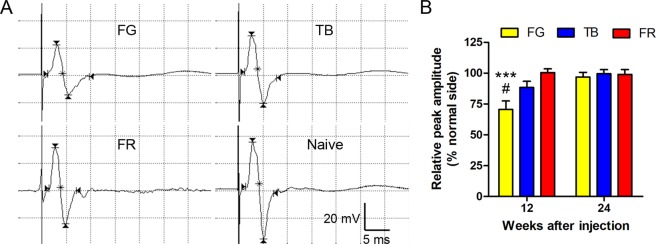
Injection of FG in the tibial nerve leads to a significant delay in recovery of compound muscle action potentials (CMAPs) in the gastrocnemius muscle. (**A**) Representative CMAP traces for each group and the contralateral naive side at 12 weeks after injection. The scale bars represent 20 mV and 5 ms, respectively. (**B**) Bar chart showing peak CMAP amplitude relative to corresponding naive side. Data are expressed as mean ± SEM (n = 6 rats each) and analyzed with two-way ANOVA followed by Bonferroni’s *post hoc* comparisons. ***P < 0.001 compared to FR, ^#^*P* < 0.05 compared to TB at the same survival time.

Electrophysiological assessment of nerve function via recording of compound muscle action potentials (CMAPs) is typically employed to evaluate recovery of nerve function; CMAP amplitude is correlated with the number of nerve fibers innervating the muscle^26,27^. We found that at 12 weeks after injection of FG into the tibial nerve, the peak amplitude of CMAPs recorded in the gastrocnemius muscle was ~70% of the contralateral normal side and significantly lower than FR and TB groups (Fig. 2A,B). However, the deficit was no longer evident 24 weeks after injection of FG (Fig. 2B). By contrast, CMAP amplitude in TB group was recovered approximately to the level in FR group by 12 weeks after injection of the tracer (Fig. 2B).

### FG-induced muscle atrophy is completely reversed at a later stage

**Figure 3:**
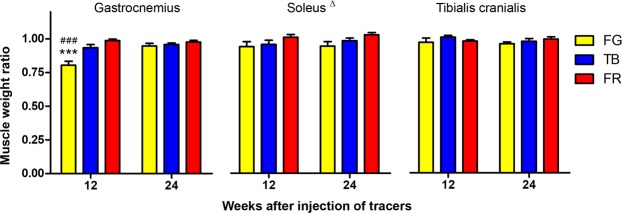
FG injected in the tibial nerve results in more prolonged atrophy of the gastrocnemius muscle than TB. Bar charts show wet weight ratio of the muscles indicated, which was calculated by dividing the muscle weight of the injected side with that of the corresponding contralateral normal side. Data are expressed as mean±SEM (n = 6 rats each) and analyzed with two-way ANOVA followed by Bonferroni’s *post hoc* comparisons. ***P < 0.001 compared to FR, ^###^*P* < 0.001 compared to TB at the same survival time. Δ indicates overall statistical significance (*P* < 0.05) for the group factor; however, no statistical significance was reached in *post hoc* comparisons.

As a common sequela of denervation, muscular atrophy is known to be completely or partially reversed as the nerve regenerate, depending on the extent of axonal regeneration and successful re-innervation of endplates^11,28^. In the present study, we found that gastrocnemius muscle, an autonomous muscle of the tibial nerve, showed significant atrophy at 12 weeks but no longer at 24 weeks after injection of FG in the tibial nerve (Fig. 3). However, the TB group did not show significant atrophy of the muscle even at 12 weeks after injection (Fig. 3). Unlike gastrocnemius, the soleus muscle did not exhibit significant difference in wet weight ratio in response in tracer exposure by 12 weeks after injection of FG, nor did the tibialis cranialis, an autonomous muscle of the peroneal nerve which served as an internal normal control (Fig. 3).

### Myelinated axons can be completely regenerated after tracer exposure

**Figure 4:**
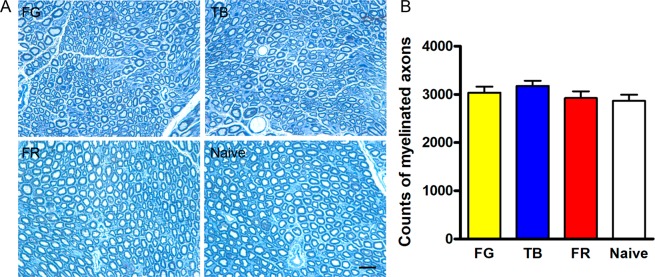
The tibial nerve distal to injection site exhibits an equal number of remyelinated axons to naive control nerve 24 weeks after injection of FG, TB or FR. (**A**) Representative photomicrographs showing semi-thin transverse nerve sections stained with toluidine blue. Bar = 20 µm. (**B**) Counts of myelinated nerve fibers in the tibial nerve. Data are expressed as mean±SEM (n = 6 rats each) and analyzed with one-way ANOVA followed by Bonferroni’s *post hoc* comparisons. No statistical significance was reached between groups.

Toluidine blue staining of semi-thin transverse sections of the tibial nerve distal to injection site showed that the nerve contained numerous myelinated axons, with thinner myelin sheath compared to the contralateral naive nerve 24 weeks after exposure to FG or TB; whereas the FR group did not show significant difference from the naive nerve in morphology (Fig. 4A). Quantification of the number of myelinated axons in the tibial nerve revealed no statistically significant difference between different tracer groups and the naive nerve (Fig. 4B), indicating that the loss of myelinated nerve fibers distal to FG or TB exposure in the peripheral nerve is completely reversible.

### TB but not FG or FR shows stable retrograde labeling in the long term

**Figure 5:**
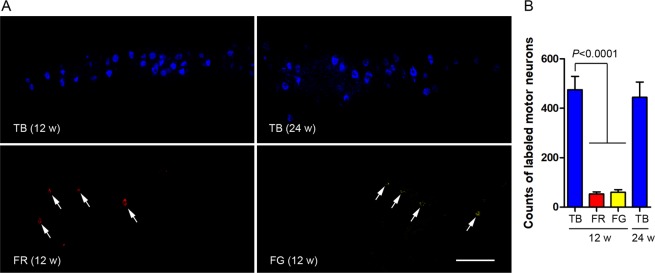
Retrograde labeling of somata of spinal motor neurons 12 and 24 weeks after injection of FG, TB or FR into the tibial nerve. (**A**) Representative confocal images of horizontal (longitudinal) section of the lumbar spinal cord showing labeling of tibial motor neuron pool. Arrows indicate somatic profiles of motor neurons in which fluorescence is markedly dimmer than TB. No fluorescence labeling of motor neurons was detectable 24 weeks after injection of FR or FG. Bar = 300 µm. (**B**) Counts of labeled tibial motor neurons. Data are expressed as mean±SEM (n = 6 rats each) and analyzed with one-way ANOVA followed by Bonferroni’s *post hoc* comparisons. There is no statistical significance in the number of TB-labeled motor neurons 12 w as compared to 24 w after tracer injection.

To learn the efficacy of retrograde labeling of neurons by the tracers on a long term basis, we quantified labeled spinal motor neurons, somata of which exhibited fluorescence of the tracer injected. We observed intense TB fluorescence in spinal motor neurons 12 and 24 weeks after injection; however, only a few motor neurons with low intensity fluorescence were visible at 12 weeks after injection of FR or FG (Fig. 5A). No FG or FR fluorescence was detectable in spinal motor neurons 24 weeks after intra-neural injection of the tracer. Quantification results showed that the number of FR or FG-labeled tibial motor neurons was only 10%-12% of that by TB at 12 weeks after injection in the tibial nerve; the number of TB-labeled motor neurons remained stable from 12 weeks to 24 weeks after injection (Fig. 5B).

## Discussion

Toxicity of tracers to neurons, including their axonal compartment, is an important domain of tracer property to be considered because in neuroregeneration and neuroanatomical studies we would expect the integrity of the fiber tract of interest. In a previous study^21^, we comprehensively characterized the function impairment resulted from injection of 5 µl of 5% FG solution or 4% TB suspension in the tibial nerve in rats in the acute phase, i.e. 3–14 days after injection. The present study extended our previous findings a step further by looking into the long-term effect of these retrograde fluorescent tracers on the peripheral system following injection into the tibial nerve. We found that as expected, intra-neural injection of FG into the tibial nerve caused prolonged motor and sensory deficits and long-term endplate denervation; but unexpectedly, these deficits appeared to be completely reversible, as the FG group did not significantly differ from TB or FR as assessed behaviorally, electrophysiologically and histopathologically 24 weeks after tracer exposure. These findings suggest that there may not be substantial neuronal death caused by FG up to 24 weeks after intra-neural injection of 2 µl of 5% solution.

In our previous study^21^, we observed degeneration of all the axons distal to injection by 5% FG, and less complete by 4% TB, after the tracer was injected at a volume of 5 µl. This indicated chemical severance of peripheral axons by FG and TB. Due to spontaneous regeneration of peripheral axons, chemically damaged axons in the tibial nerve by FG or TB injection are expected to regenerate and re-innervate the targets as long as the neuronal cell bodies maintain viability and capacity of re-growing axons. To reduce the possibility of unexpected delayed uptake of tracers by regenerated axons as a result of tracer retention at the injection site^5^, in the present study we used a smaller amount of tracers−2 µl of 5% FG solution or 4% TB suspension. We employed 2 µl of 10% FR, without inclusion of a vehicle only group, as control of no functional impairment, since we have observed that nerve histology and function did not show significant difference between injection of 5 µl of 10% FR and the same volume of saline^21^.

In the present study, we characterized the labeling efficacy of FG, TB and FR in spinal motor neurons in the long term, and we found that TB exhibited relatively stable labeling over 12 to 24 weeks after injection. By contrast, FG and FR did not show efficient neuronal labeling by 12 weeks after injection. These data are consistent with the notion that FG and FR are not suitable for long term retrograde labeling as indicated in previous studies^17^. An early study suggested that peripheral application of FG results in neuronal death based on markedly decreased number of labeled motor and sensory neurons^19^. However, significant fading of the FG fluorescence in long-survived animals^14^ may account for the decreased labeling efficacy. In fact, FG-bearing neurons can survive for up to one year and the tracer remains detectable in lysosome-like structures in neurons with anti-FG immunohistochemical staining^29–32^, consistent with its accumulation as puncta in labeled neurons as shown by our group previously^11^. The results of the present study suggest that the FG-bearing neurons are actively involved in sending/propagating electrophysiological signals, as neurofibrillary tangles-burdened neurons do^33^. The complete recovery of function is also in line with a previous study showing that intracranial injection of FG results in the degeneration of local but not retrogradely labeled neurons^20^.

In addition to local degeneration in the rat brain^20^, FG has also been shown to cause necrosis when injected in fiber tract area of the spinal cord, and the neurotoxicity appears to be related to its concentration and amount applied^18^. Interestingly, supplementation of FG solution with membrane permeabilizing detergent Triton X-100 can enhance the tracing property of FG by reducing the amount of the tracer used and the survival time required while tracing efficacy is guaranteed^18^. We previously found that the nerve tissue distal to FG injection appeared to be markedly degenerated^21^. However, the complete recovery of nerve function and absence of significant loss of myelinated nerve fibers in distal tibial nerve observed in the present study suggest that FG does not disrupt endoneurial pathways that facilities axonal regeneration. Given that tibial nerve function is recovered completely 4–8 weeks after axonotmesis or crush injury in rodents^34^, the reversible but prolonged deficit in nerve function after FG injection suggests that FG affects determinants of nerve regeneration other than the endoneurial pathway, which may include decreased growing capacity of axons and/or diminished repair capacity of Schwann cells^35,36^. The impairment could possibly be the consequence of persistence of tracer at the site of application^5^.

Unlike rapid fading of FG fluorescence *in vivo*, TB accumulated in neurons *in vivo* maintains strong fluorescence which is easily detectable up to 24 weeks, as shown in the present study and in the literature^14^. In the present study, we found that TB-induced functional deficits were recovered to a nearly normal level 12 weeks after tracer application; the functional deficits resulting from TB exposure are significantly less prolonged than by FG. This property makes TB a more suitable tracer than FG to be used in long-term neuronal tracing^9,19^. However, TB-bearing neurons may be endangered to death if additional axonal injury is inflicted, as a subsequent nerve defect injury significantly reduced the total number of TB-labeled motor neurons 3 months after tracer exposure^9^.

Local histopathological alterations induced by FG and TB are morphologically distinct from that caused by nerve transection injury^21^, suggesting that FG and TB lead to damage of other cells in addition to chemical severance of axons. In the peripheral nerve these cells may include Schwann cells, fibroblasts and endothelial cells, whereas in the central nervous system astrocytes, oligodendrocytes, microglia and endothelial cells may be involved.

Although our data showed significant reduction in the number of motor neurons labeled by FG as compared to TB, a straightforward conclusion that peripheral exposure to FG leads to neuronal loss might not be reached. To precisely identify labeled neurons which may survive a long period, a secondary tracing procedure with a second tracer before euthanasia of animals would be needed since the fluorescence of FG and FR fades markedly 12–24 weeks after application^14^, which may inflict additional and unexpected effect. Again, the complete functional recovery and similar number of myelinated axons indicate that neuronal loss induced by tracers, if any, would be minimal. It is worth noting that the complete recovery of function and regeneration of myelinated fibers may not apply to the case of tracer application in the central nervous system, where axonal regeneration is generally failed^37–40^. We would suspect that the axons distal to FG injection in the optic nerve, the spinal cord and the brain may remain degenerated and functionally impaired forever. However, this has to be investigated in future studies.

The markedly lower labeling efficacy by TB, and FR in particular, than FG observed in our previous study^21^ could be attributable to lower capability of penetrating the intact and heavily myelinated nerve fibers, which could be improved by supplementation of the tracer with a permeabilizing detergent such as dimethyl sulfoxide and Triton X-100^18,41,42^, or remedied by direct soaking of the proximal stump of a transected nerve in the tracer solution^9^. In the present study, however, extremely low labeling efficacy at 12 weeks and lack of labeling at 24 weeks after injection of FG and FR are more likely the consequence of significant fading of FG and FR fluorescence *in vivo* in the long term, since in rats survived for a short term after injection of tracers, FG exhibited highest labeling efficacy among the three tracers, ~1.5 and ~10 times higher than TB and FR, respectively^21^. In this regard, FG and FR are suitable for short-term neuronal tracing, preferably with supplementation of penetration-facilitating detergent for the latter, but their value in long-term tracing is limited.

In summary, in the present study we found that motor and sensory nerve functions were completely recovered by 24 weeks after injection of fluorescent tracers in the tibial nerve, despite a prolonged delay in the case of FG. Whereas nerve function impaired by FG and that by TB are both reversible and FR does not result in functional deficit, the stability of retrograde labeling by TB makes it a uniquely suitable fluorescent tracer, among the other two, for long-term tract tracing studies^9^.

## Materials and Methods

### Animals

Thirty-six female Sprague-Dawley rats, 3 months of age, were used in the present study. All animal procedures were carried out under the approval of Ethics Committee for Laboratory Animals at Nantong University and in accordance with US National Institutes of Health Guide for the Care and Use of Laboratory Animals published by the US National Academy of Sciences. Animals were randomized into 3 groups, i.e. FG, TB and FR (n = 12 rats each).

### Preparation of neuronal tracers

FG and FR powder (Fluorochrome LLC, Denver, CO, USA) were dissolved in sterile saline to prepare 5% and 10% solution, respectively. TB powder (Invitrogen, Carlsbad, CA, USA) was suspended in sterile saline to form 4% suspension. Aliquots of tracer solution/suspension were stored at 4 °C until used. No DMSO or other penetrating reagent was supplemented. TB suspension was mixed well immediately before each use.

### Tracer application

Rats were deeply anesthetized by intraperitoneal injection of a cocktail anesthetic solution (0.886% w/v sodium pentobarbital, 4.25% w/v chloral hydrate, 2.12% w/v magnesium sulfate, 14.25% v/v ethanol, 33.8% v/v propylene glycol) at a dose of 2.5 ml/kg body weight. The left tibial nerve was exposed under aseptic condition and injected, at 3 mm distal to the bifurcation, with 2 μl of 5% FG solution, 4% TB suspension or 10% FR solution in sterile saline using a 10-μl Hamilton syringe, which was kept in position for one additional minute so as to prevent tracer reflux. The site of injection was labeled with a 10/0 nylon suture placed in the epineurium prior to tracer injection. In order to avoid potential contamination of neighboring nerves, a piece of Parafilm^TM^ at suitable size was placed underneath to separate the tibial nerve from peroneal and sural nerves during tracer injection. The injection site was gently cleaned twice with saline-presoaked cotton gauze, and the incision was closed in layers. Animals were allowed to completely recover on a soft heating pad before returned to the home cage.

A sham control group, in which 2 μl saline only would have been intraneurally injected, was not included in the present study since we previously found that injection of 5 μl saline into the tibial nerve caused neither axonal degeneration nor functional impairment^21^.

### Walking track analysis

Rats were subjected to walking track analysis to evaluate motor nerve function at 2, 4, 12 and 24 weeks after tracer exposure, using the well established protocol^9,22^. Briefly, the plantar aspects of both hind feet were painted with non-toxic red ink, and rats were allowed to walk and pass a 42 cm × 8.2 cm track, leaving foot prints on the paper. Three parameters, namely print length (PL), toe spread (TS) and intermediate toe spread (IT), were measured from both experimental (E) and contralateral normal (N) sides. Tibial function index (TFI) was calculated with the following formula:

$$\begin{array}{ccc}{\rm{T}}{\rm{F}}{\rm{I}} & = & -37.2\times [({\rm{E}}{\rm{P}}{\rm{L}}\,-\,{\rm{N}}{\rm{P}}{\rm{L}})/{\rm{N}}{\rm{P}}{\rm{L}}]+104.4\times [({\rm{E}}{\rm{T}}{\rm{S}}\,-\,{\rm{N}}{\rm{T}}{\rm{S}})/{\rm{N}}{\rm{T}}{\rm{S}}]\\  &  & +45.6\times [({\rm{E}}{\rm{I}}{\rm{T}}\,-\,{\rm{N}}{\rm{I}}{\rm{T}})/{\rm{N}}{\rm{I}}{\rm{T}}]\,-\,\mathrm{8.8.}\end{array}$$

The value of −100 represents complete loss of tibial nerve function, and 0 for normal nerve function.

### Toe pinch reflex

Toe pinch test was performed according to the protocol described previously^24,25^ with modification. Briefly, the awake rat was restrained directly in hand and the volar aspect of the fifth toe was gently pinched with a pair of eye dressing forceps. The pain perception response to pinch was scored based on the extent of hind limb withdrawal using a three-tier scoring paradigm: 0–no response, 1–decreased response compared to normal, 2–strong and prompt withdrawal of the hind limb which is indistinguishable from the response of the contralateral normal side. The assessment was repeated three times and the highest score was selected to represent the response level. As a reference, the naive fifth toe on the contralateral side was also assessed.

### Electrophysiological examinations

A half number of rats (n = 6/group) were subjected to electrophysiological recording at 12 weeks, and the remaining rats at 24 weeks (n = 6/group) after tracer application, of CMAPs to assess motor nerve conduction and re-establishment of neuromuscular junctions^9,11^. A MYTO portable digital electromyograph recorder (EBNeuro, Italy) was employed for CMAP recording. Briefly, under deep anesthesia of the animal the sciatic nerve was exposed and stimulated with maximal electric stimuli by using a bipolar hook electrode, and CMAPs were recorded in the gastrocnemius muscle. The unipolar recording electrode and reference electrode were inserted into the muscle belly and the tendon, respectively. CMAPs of the contralateral normal side were also recorded. Relative peak CMAP amplitude of the injected side was calculated as percent of that for the contralateral normal side.

### Muscle weight ratio

Wet weight ratio of muscles can serve as a sensitive index of end-plate re-innervation efficacy following denervation^9^. Right after electrophysiological recording, rats were perfused with saline and subsequently phosphate-buffered 1.25% glutaraldehyde plus 1% paraformaldehyde. The tibial nerve distal to the injection site was excised and submerged in 4% glutaraldehyde overnight at 4 °C for histology. The gastrocnemius, soleus and tibialis cranialis muscles of both sides were precisely excised and weighed. The muscle weight ratio, i.e. wet weight of the injected side divided by that of the contralateral naive side, was calculated and compared between groups.

### Nerve histology

The tibial nerve specimens fixed with 4% glutaraldehyde were post-fixed with 1% osmium tetroxide, dehydrated in ethanol, and embedded in Epon 812 epoxy resin. Semi-thin transverse sections of the nerve 2 mm distal to the injection site were prepared and stained with toluidine blue for microscopy. Photomicographs were captured with a digital microscope (Leica Microsystems, Wetzlar, Germany) and the number of myelinated axons in the tibial nerve was manually counted.

### Laser scanning confocal microscopy

The lumbar enlargement of the spinal cord was dissected and subsequently submerged in phosphate-buffered 4% paraformaldehyde overnight at 4 °C. The specimens were dehydrated sequentially in 10%, 20% and 30% buffered sucrose solution before cryo-sectioned. The spinal cord was horizontally cut into 30-μm-thick serial sections on a cryostat. Every other sections were mounted on microscopic slides, and tracer-labeled motor neurons were visualized with an SP2 laser scanning confocal microscope (Leica Microsystems GmbH, Heidelberg, Germany), and manually counted.

### Statistical analysis

Quantitative data were subjected to one-way analysis of variance (ANOVA), two-way ANOVA or repeated measures ANOVA, where appropriate, followed by Bonferroni’s *post hoc* comparisons between groups and plotted in the GraphPad Prism 5 sofware package. *P* < 0.05 was considered statistically significant.

## Data Availability

All data and information of materials used in the present study are published in this paper. Materials are available from the corresponding authors upon reasonable request.
